# Efficient Empirical Valence Bond Simulations with GROMACS

**DOI:** 10.1021/acs.jctc.3c00714

**Published:** 2023-08-25

**Authors:** Gabriel Oanca, Florian van der Ent, Johan Åqvist

**Affiliations:** Department of Cell and Molecular Biology, Biomedical Center, Uppsala University, Uppsala SE-751 24, Sweden

## Abstract

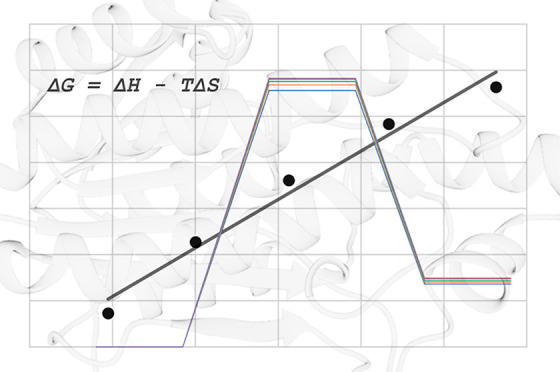

We describe a protocol to perform empirical valence bond (EVB) simulations using GROMACS software. EVB is a fast and reliable method that allows one to determine the reaction free-energy profiles in complex systems, such as enzymes, by employing classical force fields to represent a chemical reaction. Therefore, running EVB simulations is basically as fast as any classical molecular dynamics simulation, and the method uses standard free-energy calculations to map the free-energy change along a given reaction path. To exemplify and validate our EVB implementation, we replicated two cases of our earlier enzyme simulations. One of these addresses the decomposition of the activation free energy into its enthalpic and entropic components, and the other is focused on calculating the overall catalytic effect of the enzyme compared to the same reaction in water. These two examples give virtually identical results to those obtained with programs that were specifically designed for EVB simulations and show that the GROMACS implementation is robust and can be used for very large systems.

## Introduction

Biology, as we know it, would not be possible without enzymes that accelerate the chemical reactions that sustain life. The most important parameter that characterizes enzymes is their catalytic power, evaluated as the increase in the reaction rate compared to that of the uncatalyzed reaction in water. But the gain in speed does not tell us anything about how enzymes work, and if we want to control biological processes, then we must understand their internal mechanism. A step in this direction is to convert the reaction rates into free energies which can help us draw correlations between structure and functionality.^1^

In silico modeling is a powerful tool that can provide an atomistic picture of the mechanisms involved in a chemical reaction. There are several methods that can be used, which broadly fall into the following two main categories: quantum mechanics (QM) and molecular mechanics (MM). The first is generally more accurate but rather slow and restricted to systems composed of a limited number of atoms, usually at most a few hundred. The latter is less accurate, but considerably faster and allows exploration of large systems, such as enzymes, ion channels, and even entire viruses up to hundreds of millions of atoms.^2^ In these cases, long simulation times are needed to derive reliable free-energy profiles. The most efficient approach available for simulating chemical reactions in large systems is the empirical valence bond (EVB) method developed by Arieh Warshel in the ’80 s.^3^ This is a mix between QM and MM, of the QM/MM type, that uses a quantum representation of a valence bond Hamiltonian whose diagonal elements are calculated by using classical force fields.

In this paper, we present a protocol to perform EVB simulations^3–5^ using the GROMACS software.^6,7^ Since EVB relies on the free-energy perturbation (FEP) technique,^8–10^ we can take advantage of the FEP methodology already implemented in GROMACS. A typical FEP calculation in GROMACS involves transforming nonbonded interactions to calculate, for example, solvation or binding free energies. In our case, along the FEP simulation the system will be transformed from reactants to products, which implies a change not just in nonbonded but in bonded interactions as well. Since GROMACS does not offer the option to switch between bonded and nonbonded interactions between pairs of atoms, we must provide a separate topology for every FEP window. Each one of these topologies defines the right combination of reactants and products for all of the atoms involved in forming and breaking bonded interactions. We have chosen the GROMACS software since it is fast, open source, and very popular in the computational chemistry community. It is, in fact, considered to be among the fastest software for molecular dynamics (MD) simulations. This is not the first attempt to perform EVB simulations in GROMACS. A successful simulation has been reported involving a CO_2_ molecule binding to Co^1^(TPP)^−^ (cobalt *meso*-tetraphenylporphyrin) on a graphene layer^11^ but does not seem to have been generalized to other systems.

To check the validity of our protocol, we chose to replicate two of our previous computational experiments which have already been published in refs (12 and 13). The first of these is the NADH-dependent reduction of 3-oxovalerate to (*R*)-3-hydroxyvalerate catalyzed by the psychrophilic (*R*)-3-hydroxybutyrate dehydrogenase.^12^ The second case is the first proton transfer step in the conversion between dihydroxyacetone phosphate (DHAP) and glyceraldehyde-3-phosphate (GAP) catalyzed by yeast triosephosphate isomerase (TIM).^13^

## Methods

### EVB Model

The EVB method represents a chemical reaction in terms of a number of valence bond states. Any number of such states can, in principle, be used, but often a simple two-state model is considered for an elementary chemical reaction step. The system is then represented by a 2 × 2 Hamiltonian matrix
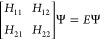
1where the ground-state energy (*E*_g_) is obtained as the lowest eigenvalue of the secular equation pertaining to the above Hamiltonian
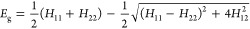
2Here, *H*_11_ and *H*_22_ are the energies of the two valence states, which are calculated using classical force fields. The off-diagonal matrix elements represent the coupling between the two states, and *H*_21_ = *H*_12_ as usual. The value of the coupling term, *H*_12_, needs to be calibrated on a reference reaction, and it has been demonstrated that it is essentially phase independent,^14^ which means that it does not change for the same reaction occurring in different surrounding media. Besides the coupling term, there is also a second parameter that must be calibrated, and it is phase-independent as well. This is the gas-phase shift, Δα, which corresponds to the (constant) difference in free energy of formation between the reacting moieties in the two states,^3^ which is not handled by regular force fields. These reference values (EVB parameters) are either evaluated from experimental data, commonly in aqueous solution, or computed by ab initio quantum calculations in water or in the gas phase^4,5^ or directly by QM/MM calculations on the enzyme.^15^ In cases where the reference reaction corresponds to a water- or gas-phase reaction, MD/EVB simulations must also be carried out in water or in the gas phase, so that *H*_12_ and Δα can be calibrated to give a free-energy profile that matches the reference values for the transition barrier and reaction free energy. The off-diagonal matrix element *H*_12_ can be represented in different ways and a common expression is given by^16^
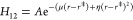
3where *r* can be the interatomic distance or the generalized reaction coordinate, ε_1_ – ε_2_ (see below). By tuning μ and η, *H*_12_ can take the form of an exponential curve, a Gaussian, or simply a flat constant (when μ and η equal zero). After calibrating the EVB reference parameters for the reaction in the given medium, these will be used without change to perform simulations of the same reaction in a more complex enzyme systems to evaluate new free-energy surfaces.

Running MD/EVB simulations to evaluate reaction free-energy profiles relies on the FEP technique, where the system is gradually shifted from the reactant state to the product state, following an effective potential energy function *V*(λ). This mapping potential is built as a linear combination between the reactant and product states, each being determined by its corresponding force field
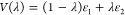
4Here, λ is an independent coupling parameter that changes in small increments from 0 to 1. The potential energies of the reactant and product states ε_1_ and ε_2_ correspond to diagonal elements *H*_11_ and *H*_22_, respectively, in eq 1 and are given by a standard MM force field. When λ = 0, the driving potential corresponds entirely to the reactant state, and at λ = 1, it will correspond to the product state. The free-energy profile from an MD/EVB simulation is calculated for the ground-state potential surface along the generalized reaction coordinate, ε_1_ – ε_2_, using the following umbrella sampling equation^4,16–18^
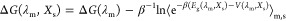
5where *X*_s_ is the generalized reaction coordinate, ε_1_ – ε_2_,^19^ which has been discretized into small bins, *s*, and β^–1^ = *k*_B_*T* (Boltzmann’s constant times the absolute temperature). *E*_g_ is the ground-state energy from eq 2, and *V*_m_ is the mapping potential *V*(λ) for a particular value of the coupling parameter λ = λ_m_ (eq 4). The free energy along the mapping potential is then calculated as
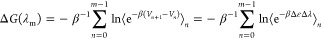
6where *V*_*n*+1_ – *V*_*n*_ is the difference in *V*(λ) between two adjacent λ-points. Due to the linear combination of eq 4, this difference can also be expressed as *V*_*n*+1_ – *V*_*n*_ = ΔεΔλ, where Δε = ε_2_ – ε_1_ and Δλ = λ_*n*+1_ – λ_*n*_, which illustrates the usefulness of keeping track of the end-point potentials and their energy gap. The brackets ⟨⟩_*n*_ represent the average value mediated by the probability , where *Q*_*n*_ is the configurational partition function for the potential *V*(λ_*n*_). In addition, the free energy on the mapping potential is calculated as an average over the forward and backward mapping directions to reduce errors due to hysteresis effects. A similar averaging applies to eq 5 by the brackets ⟨⟩_m,s_, where the difference is now taken between the ground-state energy and the mapping potential, for values given by λ_m_ that fall inside the bin *X*_s_. The final free-energy profile is then obtained by taking the weighted average of eq 5(16)
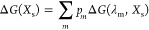
7where the sum runs over all sampling windows (λ_m_) that contribute to the bin *X*_s_, and *p*_*m*_ is the normalized statistical weight of the *m*th sampling window calculated as the ratio between the number of configurations given by the *V*(λ_m_) to the bin *X*_s_ and the total number of configurations in that bin.

### GROMACS Implementation of EVB

In most MD programs, the so-called topology file is one that defines all interatomic interactions for a system of interest, expressed as potential energies which will drive the dynamics of a simulation. These interactions can largely be grouped into two main categories, bonded and nonbonded. While a regular FEP simulation in GROMACS can handle changes in either type of potential between two different states, it cannot switch between the two. In a typical EVB simulation, the two states correspond to reactants and products and thus usually involve the breaking and forming of chemical bonds. Hence, this implies switching between bonded and nonbonded interactions for certain pairs of atoms. This includes directly bonded atoms as well as those involved in forming bond angles and torsional angles (proper and improper). As mentioned earlier, EVB relies on FEP simulations to obtain free-energy profiles, and these are performed in several windows, where for each window the coupling parameter λ is kept constant (eq 4). In our implementation of EVB, we pass a separate topology to each FEP window, where the topology has the right parameters for that particular λ-value for all atoms involved in breaking or forming chemical bonds, angles, and torsions. This is because such atoms switch between bonded and nonbonded interactions in the pure end-point potentials (λ = 0 and λ = 1).

As seen from eq 1, to calculate the ground-state energy, we must feed the diagonal elements of the EVB matrix with the energies for the two valence bond states. However, GROMACS can provide only the energy of the effective mapping potential *V*(λ) and not the energies for the two end-point states. To obtain these energies, we must recalculate them from coordinates that are saved during the FEP simulation, using the topologies that correspond to reactant state (RS) and product state (PS) (i.e., the λ = 0 and λ = 1 topology), which will give us the *H*_11_ and *H*_22_ values of the EVB matrix, respectively. The energies are then collected using the energy tool of GROMACS and analyzed by a modified version of the QFEP tool of the Q software.^1,20^ A step-by-step workflow from the beginning to the end is provided in the Supporting Information. Preparing the force field, generating the topology files, and analyzing the result was accomplished using a series of new tools that, together with the modified version of QFEP and all GROMACS input files for the protocols described below, can be found at https://github.com/gabrieloanca/gmxtools.git.

### Computational Details

**Figure 1:**
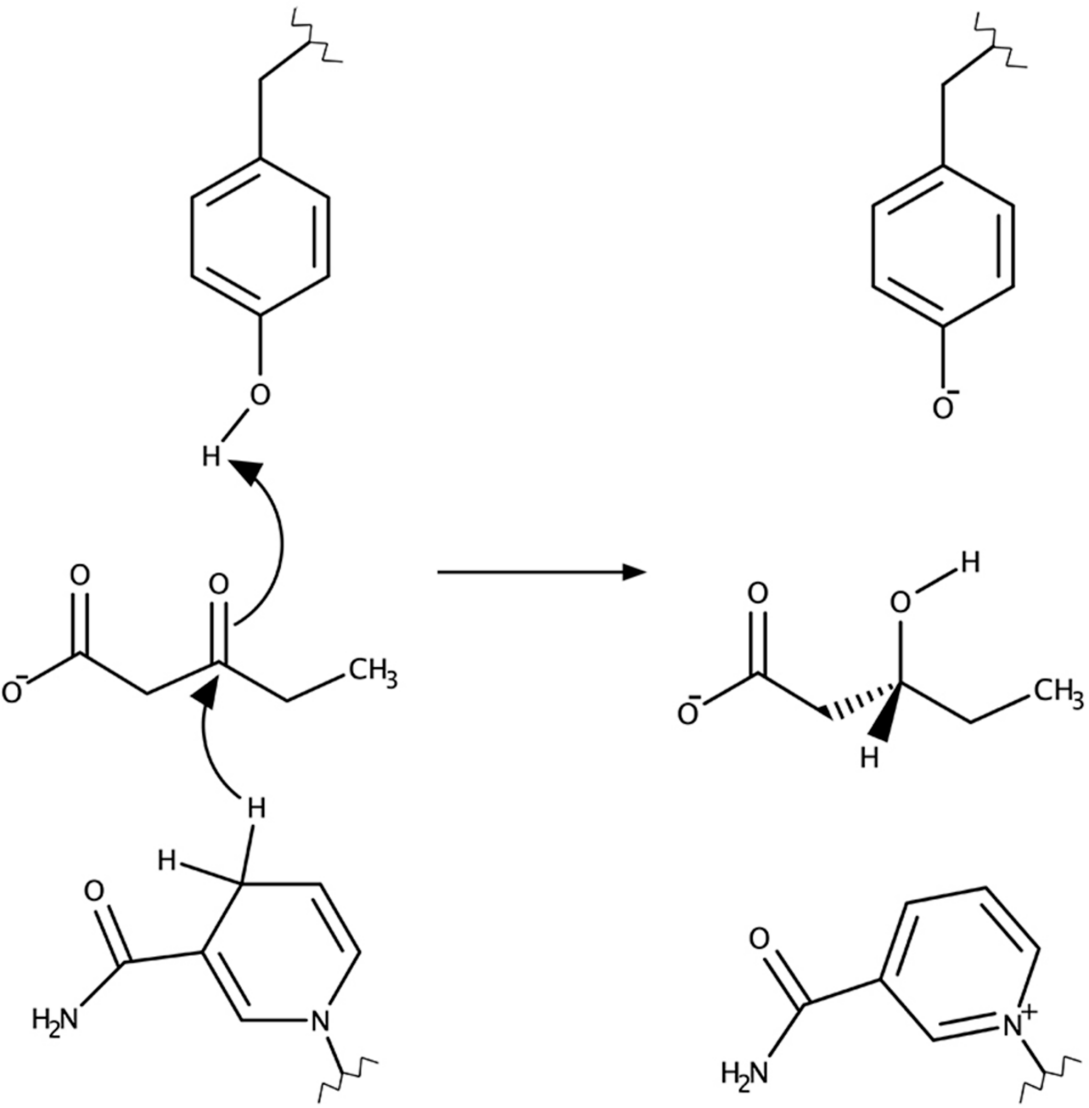
Reaction mechanism for the 3-oxovalerate substrate catalyzed by the (*R*)-3-hydroxybutyrate dehydrogenase enzyme. The concerted reaction consists of proton transfer from Tyr161 and hydride transfer from the C4 atom of the 3-carbamoyl-pyridine group of NADH cofactor to the carbonyl group of the 3-oxovalerate substrate.

To demonstrate the validity of our EVB protocol, we chose to replicate two computational experiments that have already been published by our group in refs (12 and 13). From ref (12), we simulated the conversion between 3-oxovalerate and (*R*)-3-hydroxyvalerate catalyzed by the psychrophilic (*R*)-3-hydroxybutyrate dehydrogenase from *Psychrobacter arcticus* (*Pa*HBDB) in its monomeric form. This is a concerted reaction^15^ where a hydride ion is transferred from the NADH cofactor to the substrate simultaneously with proton transfer from Tyr161 (Figure 1). The first step in preparing such a simulation consists of collecting the missing force field parameters—this is often the case whenever we are dealing with unusual compounds that are not part of the standard force field, in this case the NADH cofactor and the substrate. To this end, we first calculated the relaxed configurations and electrostatic potential (ESP) charges^21^ with the Gaussian09 software^22^ using the HF/6-31G* method,^23^ for each of the reacting moieties in the reactant and product states, including Tyr161. Each compound was optimized individually using the SMD solvation model.^24^ For tyrosine, we took only the side chain methylated at C_α_. The initial geometries for NADH and 3-oxovalerate were extracted from our previous work^12^ which utilized the 6ZZP crystal structure from the Protein Data Bank.^15^

**Figure 2:**
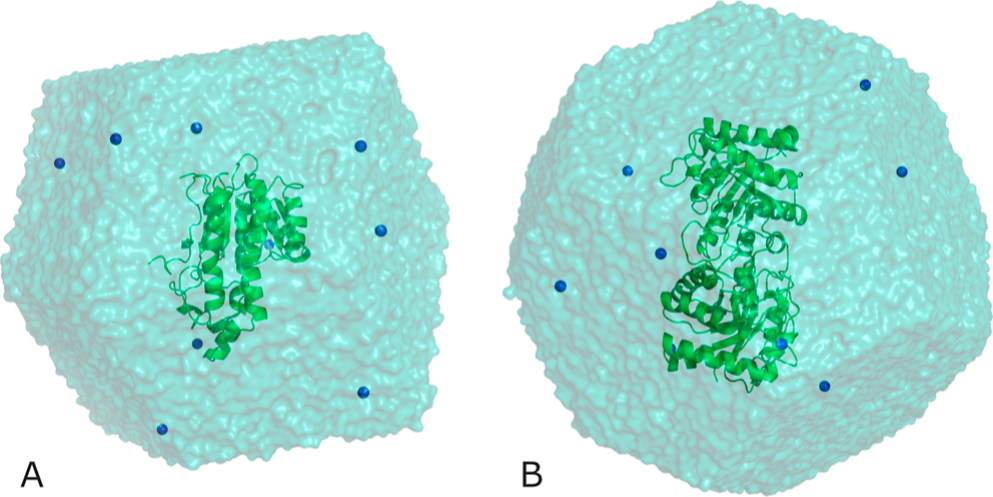
(A) Psychrophilic (*R*)-3-hydroxybutyrate dehydrogenase monomer (green ribbon) immersed in a dodecahedral box containing 23,710 water molecules. (B) TIM dimer (green ribbon) was immersed in a truncated octahedron box containing 29,301 water molecules. Blue spheres represent Na^+^ ions.

Restrained electrostatic potential (RESP) charges^25–27^ were computed from the ESP charges calculated above using AmberTools17,^28^ and the van der Waals and bonded parameters were extracted from the relaxed geometries using the ffld_server tool of Maestro2017.^29^ For the rest of the enzyme, we used the OPLS-AA/L force field^30^ together with the SPC water model^31^ for the solvent. The protonation states of ionizable residues were taken as in ref (12), from PROPKA^32^ calculations at pH 7. Together with NADH (−2) and the substrate (−1), this resulted in a total enzyme charge of −9, wherefore the system was rendered neutral by adding 9 Na^+^ counterions. All simulations were performed using the GROMACS 2022 package.^33^ The enzyme and substrate complex were enclosed in a dodecahedral periodic box with the shortest distance to the nearest wall of 2 nm and solvated with 23,710 water molecules. The size of the final system is shown in Figure 2A.

After the topologies were generated, the system was equilibrated in several steps. We first performed an *NVT* equilibration in five successive steps during which the temperature was increased from 5 to 300 K. During these equilibration steps, all heavy atoms of the protein and the Na^+^ ions were strongly restrained to their initial positions by a 200 kcal mol^–1^ Å^–2^ harmonic force constant (83,680 kJ mol^–1^ nm^–2^ in GROMACS units) in order to equilibrate the solvent, and a v-rescale thermostat was used for temperature coupling. The next four equilibration steps were performed under constant pressure, volume, and temperature (*NPT*), again raising the temperature from 5 to 300 K while also gradually decreasing the restraints from 200 to 0.5 kcal mol^–1^ Å^–2^ (209.2 kJ mol^–1^ nm^–2^), using a c-rescale barostat for pressure coupling. All nine steps consisted of 100 ps each and were followed by two additional equilibration phases, one of 10 ns at 300 K with 0.03 kcal mol^–1^ Å^–2^ (12.6 kJ mol^–1^ nm^–2^) protein heavy atom restraints and 2 kcal mol^–1^ Å^–2^ (1000 kJ mol^–1^ nm^–2^) for Na^+^ ions and, finally, one of 60 ns at 283 K and without any restraints at all.

After equilibration, we continued with the FEP simulations, which constitute the core part of the MD/EVB methodology. The FEP protocol involves a gradual change of the (mapping) potential energy by the coupling parameter λ, as given by eq 4. This change of potential will drive the system from the region of configurational space corresponding to the RS to that corresponding to the PS. In our case, this was accomplished by running a series of 51 consecutive simulations, each 20 ps long, where each simulation corresponds to a certain λ-value. Each FEP step is thus run as a separate simulation since we must use a separate topology file for each window to be able to build the correct driving potential. The same reaction simulations were repeated at five different temperatures (273, 283, 293, 303, and 313 K), and for each temperature, we ran 20 independent replicate simulations. In summary, the total length of the FEP simulation per replica was 1.02 ns, which was then repeated 100 times. At each temperature, the different replicas were the same except for the starting points, which were generated by running short 100 ps equilibrations that started from the relaxed structure for which we randomized the initial velocities. All simulations were performed with a time step of 1 fs. As in ref (12), we also applied flat bottom distance restraints between the donor and acceptor atoms involved in hydride and proton transfer with a force constant of 10 kcal mol^–1^ Å^–2^ (4187.0 kJ mol^–1^ nm^–2^) for distances larger than 3.5 Å.

**Figure 3:**
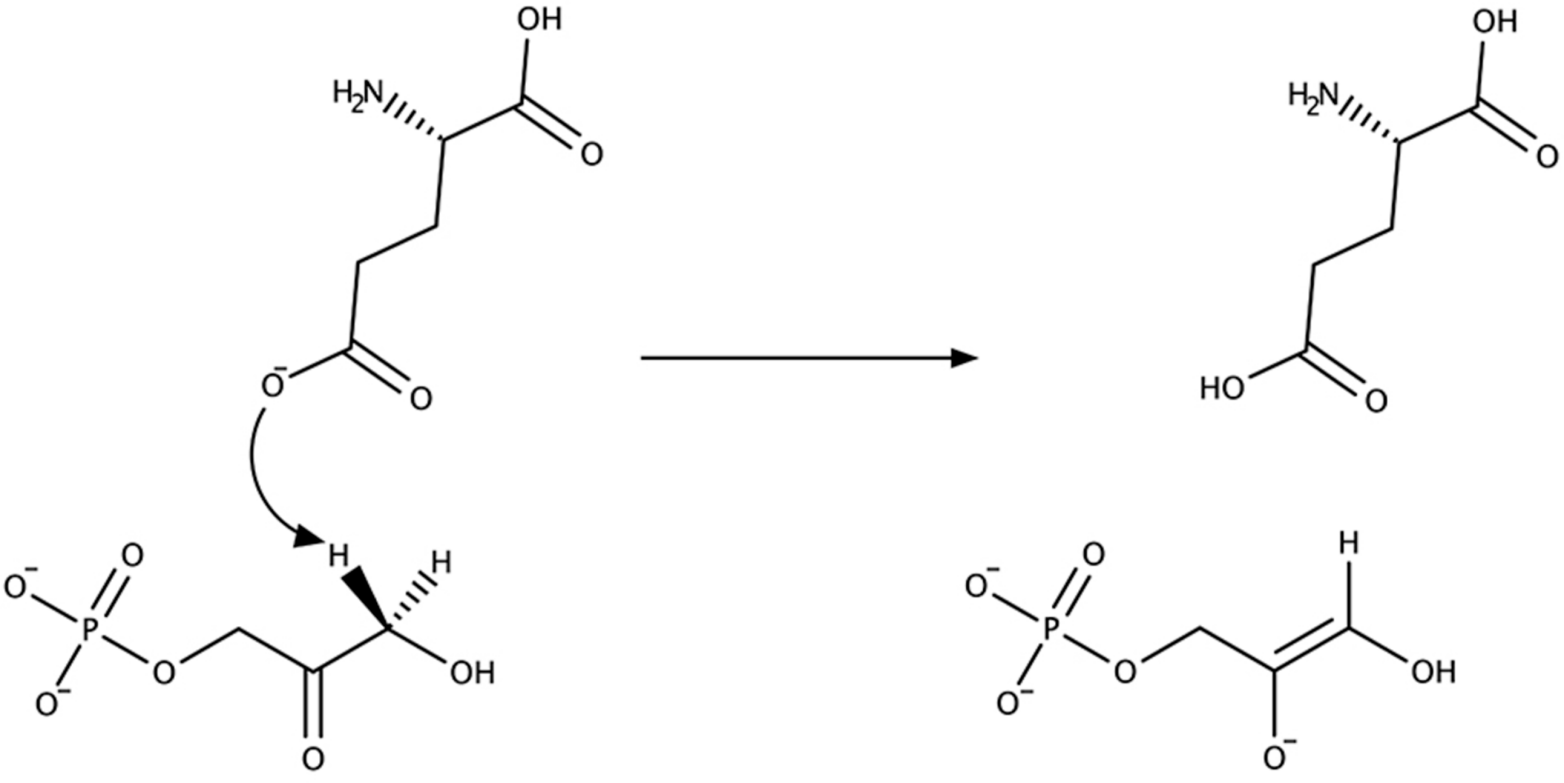
Mechanism of the initial proton transfer step in TIM where a proton is abstracted by Glu164 from the DHAP substrate.

The second experiment, which was taken from ref (13), consists of a proton transfer from the DHAP substrate to Glu164 of the yeast TIM enzyme. During this process, the ionized Glu164 abstracts a proton from DHAP which subsequently converts into GAP, via an enediolate intermediate. In our simulations, we consider the first reaction step as all internal rate constants are on the order of 10^4^ s^–1^.^34,35^ The main point with this benchmark is to analyze the overall catalytic effect compared to the uncatalyzed reaction in water, where earlier calculations gave a reduction of the activation free-energy barrier of about 12.8 kcal mol^–1^.^13,36^ The reaction mechanism of the first proton transfer step in TIM is shown in Figure 3.

The initial structure for TIM was taken from the PDB entry 1NEY.^37^ In preparing our files, we first calculated the equilibrium configuration for the substrate in the reactant and product states in Gaussian09 using the M062*X*/6-311G(d, p) method^38^ and the basis set with the SMD solvation model. From the optimized conformation, we extracted charges and van der Waals and bonded parameters using the ffld_server tool from Maestro Software, as before. It should be noted that this time we used the charges provided by Maestro and did not compute the RESP charges as we did in the previous experiment. For the rest of the enzyme, including the reacting Glu164 residue, we used the OPLS-AA/L force field^30^ and the SPC/E water model^39^ for the solvent. All ionizable residues were taken as in ref (13). which together with the −2 charge of the DHAP phosphate group resulted in a total charge of −8, wherefore the system was rendered neutral by adding 8 Na^+^ counterions. In the next step, the system was enclosed in a truncated octahedral periodic box with the shortest distance to the nearest wall of 1.2 nm and solvated with 29,301 water molecules. The size of this system is shown in Figure 2B.

After preparing the structure, topologies, and force field parameters, we used a similar equilibration protocol as in the previous experiment, with the difference that the step with 0.03 kcal mol^–1^ Å^–2^ restraints on the protein heavy atoms and 2 kcal mol^–1^ Å^–2^ on Na^+^ ions was only 1 ns long followed by another 9 ns step, where we retained the 2 kcal mol^–1^ Å^–2^ Na^+^ ion restraints (but no restraints on the protein). These last two equilibrations were both performed at 300 K. In the following steps, we also released the Na^+^ ion restraints. After equilibrating the system at the RS configuration, we continued with FEP simulations of 51 windows × 10 ps per window running from reactants to products and picked the structure at λ = 0.5, which approximately corresponds to the transition state (TS), as the starting point for the production FEP simulations. After equilibrating the structure at the TS for another 10 ns, we proceeded with simulations running from the TS toward the RS and PS, respectively. Starting FEP simulations from the TS region can potentially be more accurate by avoiding any bias toward the lower energy end-points. In our protocol, each FEP simulation consisted of 51 windows × 10 ps per window which gives a total simulation time of 510 ps. This time, the protein simulations were carried out with 30 replicas, where each replica started from a different configuration obtained by randomizing the initial velocities. During the FEP simulations, we applied flat-bottom distance restraints of 10 kcal mol^–1^ Å^–2^ for distances larger than 2 Å between the donor and the leaving proton in the RS state and between the acceptor and the incoming proton in the PS state. We also applied flat-bottom distance restraints of 10 kcal mol^–1^ Å^–2^ between the donor and acceptor for distances larger than 3 Å in both states.

For this second experiment, besides simulating the reaction inside the enzyme, we also needed to simulate the reference reaction in water, which followed the same protocol as in the enzyme with a few differences: (i) the last two equilibration steps at the RS were 100 and 500 ps long, (ii) the equilibration at the TS was 500 ps long, (iii) the FEP protocol was performed with only 20 replicas, and (iv) the backbone of the glutamate and the PO_4_ moiety of the substrate were positionally restrained with a force constant of 20 kcal mol^–1^ Å^–2^ (8368.0 kJ·mol^–1^·nm^–2^). The distance restraints between donor–acceptor and those involving the transferred proton were the same as in the enzyme, and all FEP calculations of the water and enzyme reactions were done at 300 K.

We should mention that in our implementation of EVB, as is also the case in Molaris-XG^40^ and in Q^1,20^ software (other popular software tailored for EVB simulations), whenever we have a bond breaking or forming, we substitute the regular 6–12 van der Waals potential between atoms involved in forming or breaking bonds and angles with a soft exponential repulsion potential of the following form
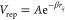
8where *r*_*ij*_ is the distance between the two atoms. This potential was passed to GROMACS as a tabulated bond of type 9, since we cannot use this type of potential for arbitrary atoms in GROMACS. In case we need to define several such repulsive potentials with different β parameters, then we must provide several tabulated potential files, one for each β. As in most force fields, bonds were expressed as harmonic potentials, except those that form or break during the reaction, in which case we used Morse potentials to ensure correct behavior. All Morse and soft repulsion parameters, together with RESP charges used in the simulations, can be found in the Supporting Information (Figures S1, S2, Tables S1, S2).

## Results and Discussion

### EVB Simulations of the HBDH Reaction with 3-Oxovalerate

In the present work, we reexamined two experiments that were previously studied by our group and published in refs (12 and 13). In the first of these, we simulated the reduction of 3-oxovalerate to (*R*)-3-hydroxyvalerate by the *Pa*HBDH enzyme in its monomeric form. The reaction involves concerted hydride ion transfer from the C4 atom of the 3-carbamoyl-pyridine group of the NADH cofactor and proton transfer from the reactive Tyr161 side chain to the substrate carbonyl group. Hence, NADH oxidizes to NAD^+^, and the Tyr161 side chain turns into a phenolate ion as a result of the reaction (Figure 1).^15^ The point of this experiment was to examine whether our GROMACS EVB implementation could correctly predict the partitioning of the activation free energy into its enthalpic and entropic components. To accomplish this task, we performed the same reaction simulations at five different temperatures, 273, 283, 293, 303, and 313 K, and calculated the free-energy components via an Arrhenius plot. Earlier QM/MM calculations on the enzyme gave predicted activation and reaction free energies of Δ*G*^⧧^ = 15.9 and Δ*G*^0^ = +3.9 kcal mol^–1^, based on exponential averaging over 10 minimum energy paths for a 25 Å radius system around the QM atoms,^15^ in good agreement with the experimentally derived value of Δ*G*^⧧^ = 16 kcal mol^–1^ at 283 K.^41^ These values of the activation barrier and reaction free energy were subsequently used to calibrate an EVB model for the tetrameric form of the enzyme in order to examine the effect of oligomerization on the thermodynamic activation parameters.^12^

**Figure 4:**
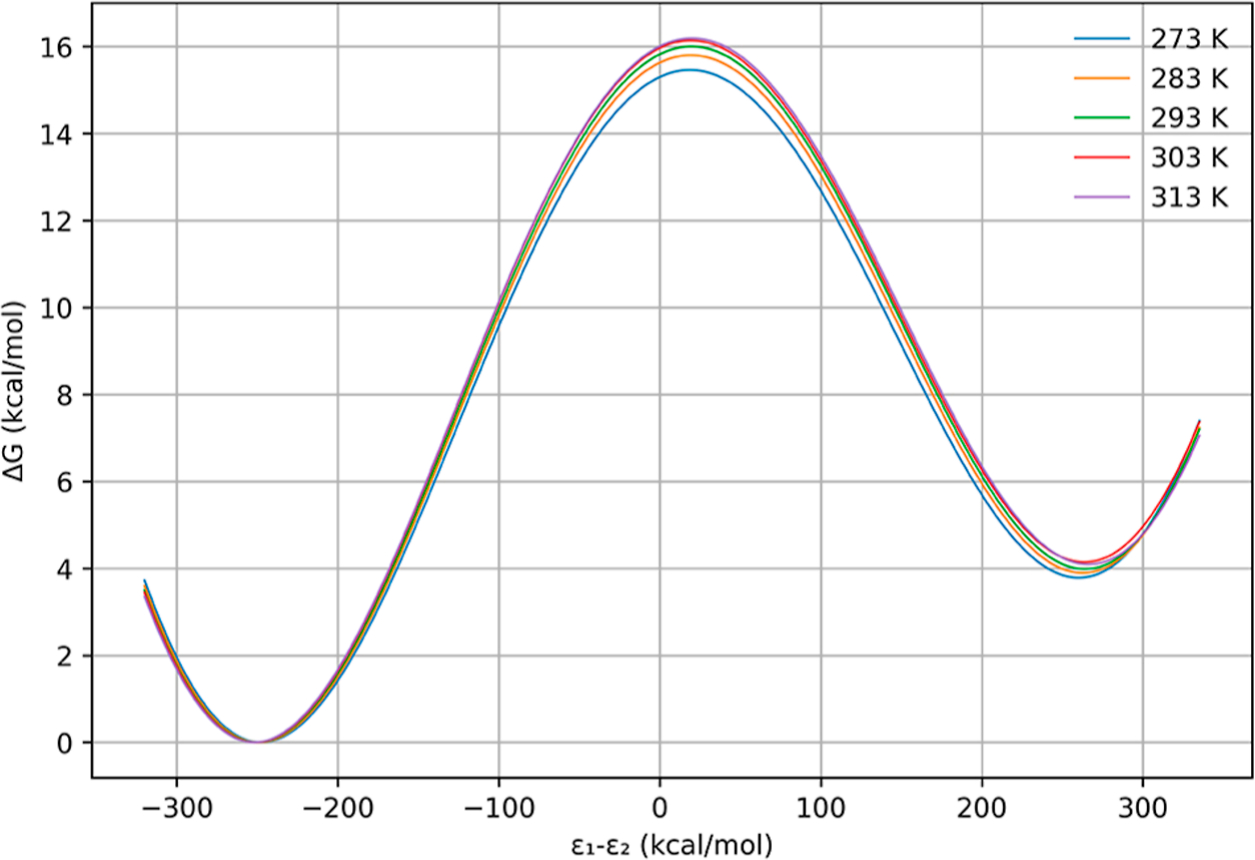
Average free-energy profiles for the reduction reaction of the 3-oxovalerate substrate catalyzed by PaHBDH at five different temperatures. ε_1_ – ε_2_ is the generalized reaction coordinate.

The main conclusion from that work was that the activation parameters were virtually unaffected by the subunit interactions and the tetramer, dimer, and monomer of *Pa*HBDH as all had activation enthalpies and entropies (*T*Δ*S*^⧧^ at 283 K) of about 10 and 5–6 kcal mol^–1^, respectively.^12^ In the present work, we can thus calibrate our EVB Hamiltonian from the GROMACS simulations at 283 K for the *Pa*HBDH monomer using the values of Δ*G*^⧧^ = 15.8 and Δ*G*^0^ = +3.9 kcal mol^–1^ obtained from the earlier MD/EVB simulations for the same system and temperature.^12^ From calibration of our EVB Hamiltonian at 283 K, we obtained the specific values of *H*_*ij*_ = 168.54 kcal mol^–1^ and Δα = 96.70 kcal mol^–1^. With this calibration, we can thus obtain Δ*G*^⧧^ and Δ*G*^0^ from MD/EVB free-energy simulations of the same reaction at all other temperatures. The results from these calculations are summarized in Table 1, as the mean over the 20 separate replicas, and the average energy profiles are shown in Figure 4.

**Table 1:** Activation Barriers (Δ*G*^⧧^) and Reaction Free Energies (Δ*G*^0^) for PaHBDH Calculated at Five Different Temperatures (kcal mol^–1^ and K) and Their Standard Deviations (SD) and Standard Errors of the Mean (SEM)

*T*	Δ*G*^⧧^	SD	SEM	Δ*G*^0^	SD	SEM
273	15.46	0.55	0.12	3.79	0.68	0.15
283	15.80	0.37	0.08	3.90	0.47	0.11
293	16.00	0.52	0.12	3.99	0.80	0.18
303	16.14	0.49	0.11	4.15	0.81	0.18
313	16.19	0.40	0.09	4.10	0.56	0.13

**Figure 5:**
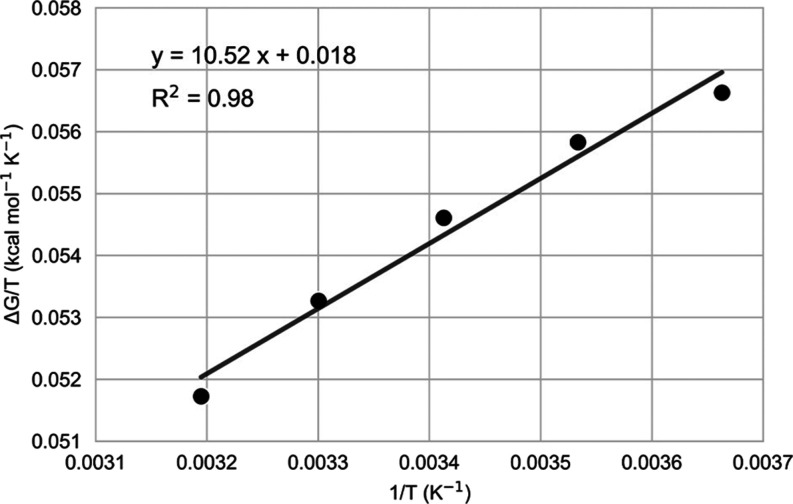
Calculated Arrhenius plot of Δ*G*^⧧^/*T* vs 1/*T* at 5 different temperatures, 273, 283, 293, 303, and 313 K. Δ*H*^⧧^ and Δ*S*^⧧^ are obtained from the linear regression equation.

From these GROMACS MD/EVB simulations, the thermodynamic activation parameters Δ*H*^⧧^ and Δ*S*^⧧^ can be reliably obtained from an Arrhenius plot of Δ*G*^⧧^/*T* versus 1/*T* (Figure 5), where the slope of the regression line corresponds to Δ*H*^⧧^, and the intercept corresponds to −Δ*S*^⧧^. The equation for the regression line is also shown in the graph and gives us Δ*H*^⧧^ = 10.52 kcal mol^–1^ and Δ*S*^⧧^ = −0.018 kcal mol^–1^ K^–1^, with a coefficient of determination *R*^2^ = 0.98. These values compare very well with the corresponding values for the monomeric form of *Pa*HBDH reported in ref (12), which were Δ*H*^⧧^ = 10.30 kcal mol^–1^ for enthalpy and Δ*S*^⧧^ = −0.020 kcal mol^–1^ K^–1^ for entropy. The values predicted for *T*Δ*S*^⧧^ at 283 K are thus −5.1 kcal mol^–1^ from the present simulations compared to −5.5 kcal mol^–1^ in ref (12).

### EVB Simulations of Proton Transfer in TIM

The second experiment deals with MD/EVB simulations of the first proton transfer step in TIM, as reported earlier.^13,36^ This case is different than the previous one and arguably more important since here we directly compare the effect of the enzyme on the reaction energetics of the uncatalyzed reference reaction in water. This is also the most common application of EVB calculations, which allows the catalytic effect to be quantified for a given enzyme, and its structural origin may thus be elicited. TIM catalyzes the conversion of DHAP into GAP through a series of four proton transfers.^34,35^ Here we just consider the first step in order to validate our EVB implementation in GROMACS by comparison to the results of ref (13).

The reaction is shown in Figure 3 and involves proton transfer from the DHAP substrate to the negatively charged carboxylate side chain of Glu164, which leads to the formation of an enediolate intermediate. The key point here is that we need to calibrate the EVB parameters *H*_12_ and Δα from simulations of the same uncatalyzed process in aqueous solution at 300 K. We thus took the same target values for the water reaction calibration as in ref (13), Δ*G*^⧧^ = 23.6 kcal mol^–1^ and Δ*G*^0^ = 18.8 kcal mol^–1^ (Table 2). This calibration yielded values for *H*_*ij*_ and Δα of 92.66 and 85.91 kcal mol^–1^, respectively, which thus reproduce the target free energies exactly for the uncatalyzed reference reaction.

**Table 2:** Activation Barriers (Δ*G*^⧧^) and Reaction Free Energies (Δ*G*^0^) for the Proton Transfer Step in TIM and in Water (kcal mol^–1^) and Their Standard Deviation (SD) and Standard Error of the Mean (SEM)

system	Δ*G*^⧧^	SD	SEM	Δ*G*^0^	SD	SEM
water	23.60	0.61	0.14	18.80	0.86	0.19
TIM	13.34	0.86	0.16	2.64	1.12	0.20

**Figure 6:**
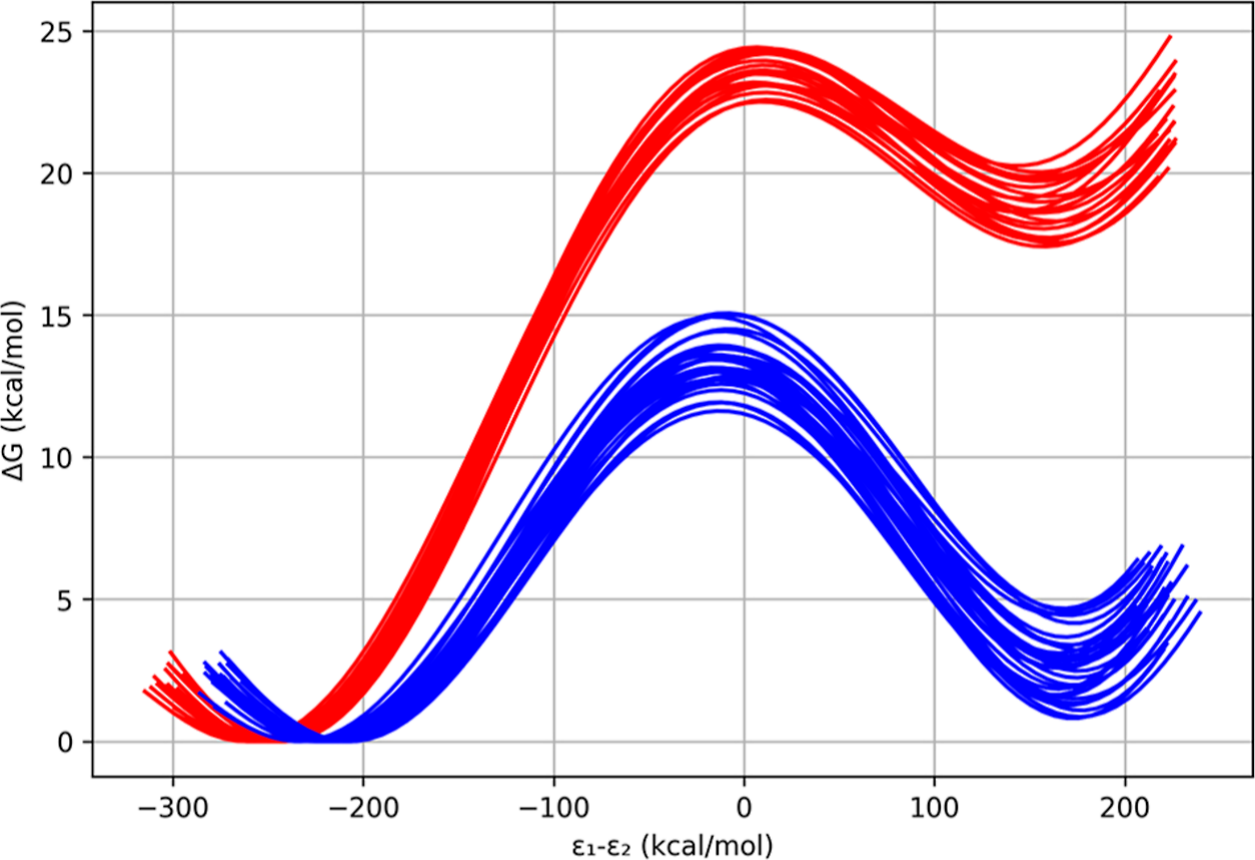
Calculated free-energy profiles for proton abstraction from DHAP in water (red) and in the TIM enzyme (blue). ε_1_ – ε_2_ is the generalized reaction coordinate.

These values were then also used in the MD/EVB simulations of the enzyme catalyzed reaction, and the resulting free-energy profiles for the water and enzyme reactions are shown in Figure 6 and are summarized in Table 2. The large catalytic effect of the enzyme is immediately evident and is primarily due to the very efficient stabilization of the high-energy enolate intermediate.^13,36^ The GROMACS MD/EVB simulations yield a reduction of the activation free energy Δ*G*^⧧^ from 23.6 kcal mol^–1^ in water to 13.3 kcal mol^–1^ in TIM, which is very similar to the value of Δ*G*^⧧^ = 12.8 kcal mol^–1^ obtained in ref (13). Hence, we can conclude that the catalytic effect in TIM is also accurately captured by the MD/EVB simulations with our GROMACS implementation.

## Conclusions

We have presented here an EVB implementation with the GROMACS software^6,7^ and validated this by repeating simulations carried out earlier for two different enzymes with the Q program.^1,20^ Reaction free-energy profiles were thus calculated for the catalytic reactions of a hydroxybutyrate dehydrogenase and TIM as well as for the uncatalyzed TIM reaction in water. In the first case, the focus was on examining whether the enthalpic and entropic contributions to the activation free energy could be reproduced by our protocol, which involves MD/EVB simulations at a series of different temperatures to obtain the computational Arrhenius plots. The conclusion here is that the present implementation gives very similar results to those obtained earlier, despite the fact that the simulation systems are significantly different. The same conclusion was reached for the simulations of the TIM catalyzed reaction, where the large catalytic effect of the enzyme was accurately reproduced. These two examples also clearly demonstrate the power of the EVB method for quantitative modeling of enzyme catalyzed reactions.

The advantage of being able to use GROMACS for EVB calculations is that it is among the fastest MD programs and allows for simulations of very large systems. In particular, the Ewald-type lattice summation^42^ of long-range electrostatics scales favorably with system size and is a key reason for the efficiency. With the spherical boundary systems usually used in EVB simulations,^1,20,40^ crystal lattice summation cannot be used, and the local reaction field (LRF) multipole expansion method^43^ that is usually employed becomes very costly for large systems unless it is combined with interaction cutoffs. With LRF, the system is divided into neutral groups (assemblies of three or four atoms), but its computational cost still increases as *N*^2^, with *N* being the number of neutral groups in this case, and its main advantage arises from the fact that the long-range potential changes very slowly, which does not require being updated too often (usually between 20 and 50 time steps). Overall, the grouping of atoms and the slow change in long-range potential decrease the computational complexity by 2 orders of magnitude. On the other hand, the Ewald summation scales as *N* log(*N*) as it performs the summation in Fourier space. Most questions regarding enzyme catalysis can, however, be reliably addressed using the smaller spherical systems, but one can clearly envisage cases where size matters more, and the EVB implementation presented here can thus be very useful.

As is evident from our calculation scheme, there are some interesting program-specific problems that are encountered. In GROMACS and several other MD programs, a pair of atoms cannot be bonded (or angled) in one state of a FEP calculation (with no nonbonded interactions) and nonbonded in the other state (with no bonded interaction). This is because the FEP machinery in these programs was simply not designed for breaking chemical bonds and replacing them by nonbonded interactions. This is, however, a key element of the EVB method that needs to be there. Another somewhat annoying problem is that several programs, including GROMACS, do not actually express the potential energy functions at intermediate λ-values as a clean linear combination of the two end-states (eq 4). An obvious advantage with using eq 4 is that both potential energies and forces only need to be calculated repeatedly for the two end-states during the FEP trajectories and are then scaled by λ to achieve the relevant intermediate values of *V*(λ). GROMACS and several other programs instead scale the individual interaction parameters (*k*_b_, b_0_ for harmonic bonds, *k*_θ_, θ_0_ for angles, etc.) themselves by λ rather than the total potential energy. Hence, eq 4 and the right-hand side of eq 6 are then not valid, and there is therefore little meaning in keeping track of the end-point energies during simulations of the intermediate states. This is thus the reason we need to recalculate the end-point energies from the generated trajectories at all λ-values, as they play a central role in the EVB method (eq 2). Nevertheless, with the procedures now in place for generating all energetic data needed for EVB calculations with GROMACS, we believe that this will provide a useful tool for people who may want to examine chemical reactions with this methodology. The scripts necessary for preparing the parameters and the topologies for an EVB simulation in GROMACS as well as the input files for the protocols described in this paper can be found at https://github.com/gabrieloanca/gmxtools.git.

## Supporting Information Available

Step-by-step workflow with command lines and MD parameters of the EVB atoms for the experiments presented in this paper, including the electrostatic charges (PDF)
