# Supplementary material for: Efficient Empirical Valence Bond Simulations with GROMACS
Source: J Chem Theory Comput. 2023 Aug 25;19(17):6037–45. doi: 10.1021/acs.jctc.3c00714 (PMC10500987; doi:10.1021/acs.jctc.3c00714)
Supplement: Supplementary file 1 — ct3c00714_si_001.pdf [file ct3c00714_si_001.pdf]

# Supporting Information for:

## Efficient Empirical Valence Bond Simulations with GROMACS

Gabriel Oanca,* Florian van der Ent, Johan Åqvist*

*Department of Cell and Molecular Biology, Uppsala University, Biomedical Center,*

*SE-751 24 Uppsala, Sweden*

*Corresponding authors

e-mail: gabriel.oanca@icm.uu.se, johan.aqvist@icm.uu.se

## The workflow

The workflow presented below will guide you through the process of running an Empirical Valence Bond (EVB) simulation [1-3] using GROMACS software. [4, 5] As an example, we will consider the simulation for the *Pa*HBDH system. The force field, the topologies, and other intermediary files can be found at https://github.com/gabrieloanca/ JCTC_2023.git. The tools that we developed in our laboratory can be downloaded from https://github.com/gabrieloanca/gmxtools.git. To use these tools as indicated below, after downloading add them to the **PATH** environment variable. These commands will execute on a LINUX terminal. To obtain the full list of tools, type "gmxtools" in terminal. For help, type "-h" after a tool's name. The GROMACS input files for the protocol indicated in steps II and III can also be found at the latter link. In this report we will consider the folders and file names as there.

### I Preparing the system and building the topologies

1. Calculate the relaxed geometries and ESP charges with Gaussian09[6] for the reacting moieties which include the 3-oxovalerate substrate, nicotinamide adenine dinucleotide cofactor (NADH) and tyrosine 161 (Tyr161) in reactant state (RS) and product state (PS) . We will obtain the following files for RS: **3ov.log** (substrate), **nah.log** (NADH) and **twn.log** (Tyr161); and for PS we will have: **hov.log** (substrate), **nad.log** (NADH), and **twm.log** (Tyr161).

2. Calculate the RESP charges with antechamber from AmberTools17.[7] The command below will repeat for each output file from Gaussian, but for convenience we will show an example only for one of them.

```
antechamber -fi gout -i 3ov.log -fo ac -o 3ov.ac -c resp -nc -1
```

3. Extract the pdb from the gaussian output file.

```
antechamber -fi gout -i 3ov.log -fo pdb -o 3ov.pdb -rn 3OV
```

4. Calculate the ffld_server[8] parameters.

```
ffld_server -version 14 -print_parameters -ipdb 3ov.pdb > 3ov.ffld
```

5. Convert the ffld_server parameters into GROMACS' OPLS-AA force field format. For 3-oxovalerate substrate we will obtain the following files: **3ov_atomtypes.opls**, **3ov_vdw.opls**, **3ov_bonds.opls**, **3ov_angles.opls**, **3ov_torsions.opls**, and **3ov_impropers.opls.**

```
ffld2gmx.py -n 3ov -f 3ov.ffld -a 3ov.ac
```

6. Add the content from **3ov_atomtypes.opls** to **atomtypes.atp**, add **3ov_vdw.opls** to **ffnonbonded.itp**, and **3ov_bonds.opls**, **3ov_angles.opls**, **3ov_torsions.opls**, and **3ov_impropers.opls** to **ffbonded.itp** file of GROMACS' force field. (For convenience, you can write only the parameters for RS in **ffbonded.itp** file and substitute the bonding types for the PS atom types inside **ffnonbonded.itp** with the corresponding bonding types for RS).

7. Add dummy atom types for all EVB atoms in **ffnonbonded.itp** and **atomtypes.atp** files (see the files at the address indicated above).

8. Build the corresponding residue inside **aminoacids.rtp** file (see 3OV residue inside **aminoacids.rtp**); we must build only the residues corresponding to RS.

NOTE: For now, we must do this step manually; in future versions we will consider building .itp files which will allow us to skip the steps from 6 to 8.

9. Build GROMACS topology.

```
gmx pdb2gmx -f hbdh.pdb -o hbdh-start.pdb -water spc -merge all
```

10. Build the periodic box.

```
gmx editconf -f hbdh-start.pdb -o hbdh-box.pdb -c -d 2 -bt dodecahedron
```

11. Solvate the system.

```
gmx solvate -cp hbdh-box.pdb -cs spc216.gro -o hbdh-solv.pdb -p topol.top
```

12. Add ions.

gmx grompp -f restart.mdp -c hbdh-solv.pdb -p topol.top -o ions.tpr -maxwarn 2

gmx genion -s ions.tpr -o hbdh -ions.pdb -p topol.top -pname NA -nname CL -neutral

13. Add the restraints at the end of the topology file (see topologies at the address indicated above).

14. Define the EVB atoms, Morse bonds, soft-repulsion, and restraints inside **qmatoms.dat** file.

15. Build the topologies for EVB; there will be 51 topologies for a 51 frames FEP simulation.

gmx4evb.py -f 51 -r 3ov nah twn -p hov nad twm

16. Generate the position restraints files (these files will correspond to the restraints mentioned in step 13). The following example will build a file with 209.2 kJ mol$^{-1}$ nm$^{-2}$ (0.5 kcal mol$^{-1}$ Å$^{-2}$) on EVB atoms and 12.6 kJ mol$^{-1}$ nm$^{-2}$ (0.03 kcal mol$^{-1}$ Å$^{-2}$) for the rest of the protein.

genposre.py --r1 209.2 --r2 12.6

17. Generate tabulated potential files for soft-repulsion interactions. The soft-repulsion potential has the following expression:

$$V = Ae^{-\beta r_{ij}}$$

and it is passed to GROMACS as a tabulated bond of type 9. These files will contain three columns with discrete data corresponding to the following functions: $r_{ij}$, $e^{-\beta r_{ij}}$, $\beta e^{-\beta r_{ij}}$.

## II Equilibration and production run

1. Copy the position restraints files, the tabulated potential files, topol_000.top and the modified force field directory into the equilibration folder (**equil**) and then run the equilibration steps sequentially.

2. Copy the last equilibrated .gro file inside the production folder (**fep/rep_000**) together with the tabulated potential files, all topology files, and the force field directory. First run **restart.mdp** file which further equilibrates the system starting from randomized velocities - this way we can provide a different starting point to each FEP replica. Then submit the FEP frames sequentially, passing to each frame its corresponding topology (topol_000.top to fep_000.mdp, topol_001.top to fep_001.mdp and so on).

## III Extracting the valence energies and building the EVB profile

1. Move all the trajectory (.trr) files to **rerun** folder (**fep/rep_000/rerun**). Copy also the initial .gro file, the force field directory, the tabulated potential files, **topol_000.top**, **topol_050.top** (fist and last topology), and **evbless.top**. This last topology file is a special topology where all the EVB atoms have been converted into dummies. Then rerun all the FEP frames on the coordinates from the trajectory files using the topology corresponding to RS (**topol_000.top**), then with the PS topology (**topol_050.top**) and one last time using **evbless.top** topology.

2. Extract the potential energies from each rerun. To this end, you can use the bash script **get_ene.sh** found inside the **fep** folder. Inside this file, you must give the correct number to **nosys** and **noless** variables which correspond to the numbers passed to the **energy** tool of GROMACS to extract the potential energy from the rerun with the RS and PS topologies (**nosys**) and with **evbless.top** topology (**noless**). At the end of this process, we will obtain two folders, **sysA** and **sysB**, with files containing the extracted potential energies.

3. Calculate the valence energies $H_{11}$ and $H_{22}$ (see main text) and write them into a format appropriate for **qfep** analysis.

```
gmx2qfep.py -f 51
```

4. Prepare the input file for **qfep** (see **qfep.inp** file inside **fep** folder). For Q users, this file is the same as for Q simulations.[9, 10]

```
qfep5_gmx < qfep.inp > qfep.out
```

The output (**qfep.out**) is the same as for Q simulations - the EVB profile can be found at the end of this file, under the section "Part 3", and can be visualized by plotting the columns "energy gap" vs. "<dGg norm>".

**Table S1.** EVB parameters for the reaction of 3-oxovalerate substrate catalyzed by (R)-3-hydroxybutyrate dehydrogenase from *Psychrobacter arcticus* enzyme (*Pa*HBDB). The parameters include the RESP charges for the EVB atoms in reactant (RS) and product (PS) states, soft exponential repulsion for the atoms that form or break chemical bonds and Morse potential parameters. The EVB atoms include the side chain of tyrosine 161 (TYR), the 3-carbamoyl-pyridine group of NADH (NAH) and the entire substrate (3OV). The atom names are the same as in topology and are shown in Figure S1. The residue names are the same as in topology as well.

| *Moiety* | *Atom* | *RESP charges ($e^-$)* | |
|---|---|---|---|
| | | RS | PS |
| *TYR* | CB | 0.104314 | 0.137870 |
| | HB | 0.001268 | -0.015654 |
| | CG | -0.078705 | -0.203621 |
| | CD1 | -0.132965 | -0.089370 |
| | HD1 | 0.176128 | 0.158229 |
| | CD2 | -0.132965 | -0.089370 |
| | HD2 | 0.176128 | 0.158229 |
| | CE1 | -0.414256 | -0.545642 |
| | HE1 | 0.214901 | 0.170448 |
| | CE2 | -0.414256 | -0.545642 |
| | HE2 | 0.214901 | 0.170448 |
| | CZ | 0.515794 | 0.710039 |
| | OH | -0.708780 | -1.000310 |
| | HH | 0.477225 | 0.486870 |
| *3OV* | O1 | -0.904917 | -0.907567 |
| | C1 | 0.956537 | 0.977222 |
| | O2 | -0.904917 | -0.907567 |
| | C2 | -0.330350 | -0.420209 |

| | | | |
|---|---|---|---|
| | C3 | 0.641585 | 0.507717 |
| | O3 | -0.687840 | -0.859251 |
| | C4 | 0.006704 | 0.012929 |
| | H1 | 0.095219 | 0.091895 |
| | H2 | 0.095219 | 0.091895 |
| | H3 | 0.021398 | -0.007508 |
| | H4 | 0.021398 | -0.007508 |
| | C5 | -0.142341 | -0.106123 |
| | H5 | 0.044102 | 0.021892 |
| | H6 | 0.044102 | 0.021892 |
| | H7 | 0.044102 | 0.021892 |
| *NAH* | C1 | -0.274708 | 0.015414 |
| | H1 | 0.222078 | 0.215625 |
| | C2 | -0.237512 | -0.207470 |
| | H2 | 0.146185 | 0.220808 |
| | C3 | 0.264104 | 0.052289 |
| | H3 | -0.015344 | -0.018470 |
| | H4 | -0.015344 | 0.190475 |
| | C4 | -0.323854 | -0.144760 |
| | C5 | 0.977668 | 0.949456 |
| | O1 | -0.784769 | -0.725673 |
| | N1 | -1.110047 | -0.990441 |
| | H5 | 0.491789 | 0.487375 |
| | H6 | 0.491789 | 0.487375 |
| | C6 | -0.027435 | -0.028633 |
| | H7 | 0.194457 | 0.279830 |
| | N2 | 0.000943 | 0.198330 |
| *soft exponential repulsion* | | | |

| *Atom pair* | *C (kJ/mol)* | *β (nm$^{-1}$)* |
|---|---|---|
| OH(TYR)- HH(TYR) | 1046.00 | 20.00 |
| O3(3OV)- HH(Tyr160) | 1046.00 | 20.00 |
| C3(NAH)- H3(NAH) | 1046.00 | 20.00 |
| C3(3OV)- H3(NAH) | 1046.00 | 20.00 |

*Morse potential*

| *Atom pair* | *D (kJ/mol)* | *β (nm$^{-1}$)* | *$r_0$ (nm)* |
|---|---|---|---|
| OH(Tyr)- HH(Tyr) | 1028.33 | 15.0 | 0.0945 |
| O3(3OV)- HH(Tyr) | 1028.33 | 15.0 | 0.0945 |
| C3(NAH)- H3(NAH) | 632.25 | 15.0 | 0.1090 |
| C3(3OV)- H3(NAH) | 632.25 | 15.0 | 0.1090 |

*EVB matrix calibrated parameters*

| *off-diagonal (kcal/mol)* | *gas phase shift (kcal/mol)* |
|---|---|
| 168.54 | 96.70 |

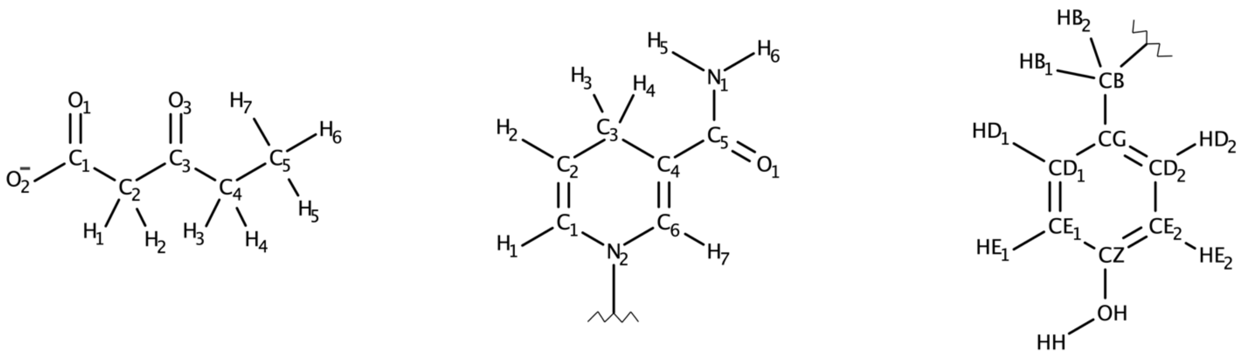


3OV NAH TYR

**Figure S1.** Reacting moieties with atom names. The atom names correspond to those in Table S1 and in the topology.

**Table S2.** EVB parameters for the reaction of dihydroxyacetone phosphate (DHAP) substrate catalyzed by triosephosphate isomerase (TIM) enzyme. The parameters include the ffld_server generated charges for the EVB atoms in reactant (RS) and product (PS) states, soft exponential repulsion for the atoms that form or break chemical bonds and Morse potential parameters. The EVB atoms are shown with labels in Figure S2. The labels in this table correspond to those in Figure S2 and in topology. The residue names are the same as in topology as well.

| *Moiety* | *Atom* | *RESP charges ($e^-$)* | |
|---|---|---|---|
| | | RS | PS |
| *GUW* | CG | -0.220 | -0.120 |
| | HG | 0.060 | 0.060 |
| | CD | 0.700 | 0.520 |
| | OE1 | -0.800 | -0.440 |
| | OE2 | -0.800 | -0.530 |
| *SUB* | C9 | 0.47 | 0.2225 |
| | O10 | -0.47 | -1.2225 |
| | C11 | 0.145 | 0.1075 |
| | O12 | -0.683 | -0.6405 |
| | H13 | 0.418 | 0.418 |
| | H14 | 0.06 | 0.115 |
| | H15 | 0.06 | 0.450 |
| *soft exponential repulsion* | | | |
| *Atom pair* | | *C (kJ/mol)* | *β ($nm^{-1}$)* |
| OE2(GUW)- H15(SUB) | | 1108.76 | 25.00 |
| H15(SUB)- C11(SUB) | | 1046.00 | 25.00 |
| OE2(GUW)- C11(SUB) | | 84429.10 | 30.0 |

| | | |
|---|---|---|
| C9(SUB)- H15(SUB) | 4639.01 | 30.0 |
| O12(SUB)- H15(SUB) | 4639.01 | 30.0 |
| H14(SUB)- H15(SUB) | 437.65 | 30.0 |
| CD(GUW)- H15(SUB) | 4639.01 | 30.0 |

| *Morse potential* | | | |
|---|---|---|---|
| *Atom pair* | *D (kJ/mol)* | *β ($nm^{-1}$)* | *$r_0$ (nm)* |
| C11(SUB)- H15(SUB) | 410.00 | 15.0 | 0.1090 |
| OE2(GUW)- H15(SUB) | 460.00 | 15.0 | 0.0945 |

| *EVB matrix calibrated parameters* | |
|---|---|
| *off-diagonal (kcal/mol)* | *gas phase shift (kcal/mol)* |
| 93.25 | 86.71 |

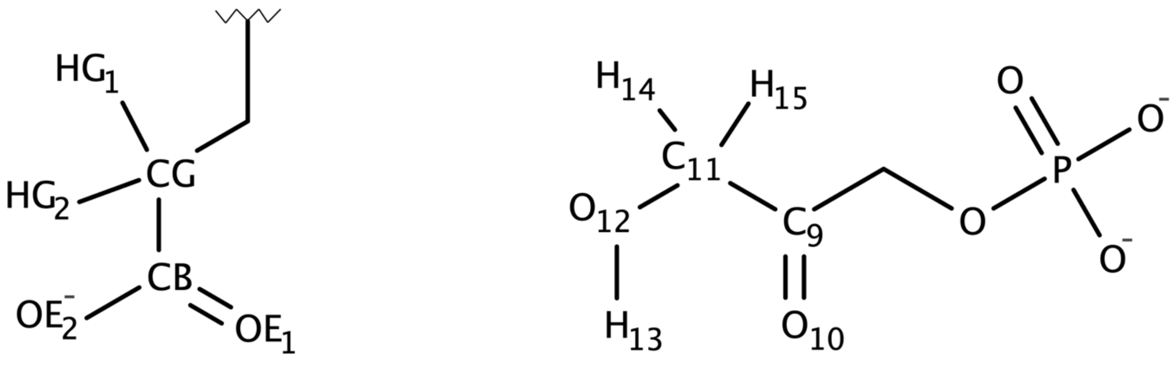


GUW SUB

**Figure S2.** Reacting moieties with atom names. The atom names correspond to those in Table S2 and in the topology.

## References

(1) Warshel, A., Weiss, R. M. An Empirical Valence Bond Approach for Comparing Reactions in Solutions and in Enzymes. *J. Am. Chem. Soc.* **1980**, *102*, 6218-6226.

(2) Warshel, A. Computer Modeling of Chemical Reactions in Enzymes and Solutions. John Wiley & Sons: New York, 1991.

(3) Åqvist, J., Warshel, A. Simulation of Enzyme-Reactions Using Valence-Bond Force-Fields and Other Hybrid Quantum-Classical Approaches. *Chem. Rev.* **1993**, *93*, 2523-2544.

(4) Lindahl, E., Hess, B., van der Spoel, D. GROMACS 3.0: A Package for Molecular Simulation and Trajectory Analysis. *J. Mol. Model.* **2001**, *7*, 306-317.

(5) Abraham, M. J., Murtola, T., Schulz, R., Páll, S., Smith, J. C., Hess, B., Lindahl, E. Gromacs: high performance molecular simulations through multi-level parallelism from laptops to supercomputers. *SoftwareX* **2015**, *1-2*, 19-25.

(6) Frisch, M. J., Trucks, G. W., Schlegel, H. B., Scuseria, G. E., Robb, M. A., Cheeseman, J. R., Scalmani, G., Barone, V., Mennucci, B., Petersson, G. A., Nakatsuji, H., Caricato, M., Li, X., Hratchian, H. P., Izmaylov, A. F., Bloino, J., Zheng, G., Sonnenberg, J. L., Hada, M., Ehara, M., Toyota, K., Fukuda, R., Hasegawa, J., Ishida, M., Nakajima, T., Honda, Y., Kitao, O., Nakai, H., Vreven, T., Montgomery, J. A., Peralta, J. E., Ogliaro, F., Bearpark, M., Heyd, J. J., Brothers, E., Kudin, K. N., Staroverov, V. N., Keith, T., Kobayashi, R., Normand, J., Raghavachari, K., Rendell, A., Burant, J. C., Iyengar, S. S., J. Tomasi, Cossi, M., Rega, N., Millam, J. M., Klene, M., Knox, J. E., Cross, J. B., Bakken, V., Adamo, C., Jaramillo, J., Gomperts, R., Stratmann, R. E., Yazyev, O., Austin, A. J., Cammi, R., Pomelli, C., Ochterski, J. W., Martin, R. L., Morokuma, K., Zakrzewski, V. G., Voth, G. A., Salvador, P., Dannenberg, J. J., Dapprich, S., Daniels, A. D., Farkas, O., Foresman, J. B., Ortiz, J. V., Cioslowski, J., Fox, D. J. *Gaussian 09*, Revision E.01; Gaussian, Inc.: Wallingford CT, 2013.

(7) Case, D. A.; Cerutti, D. S.; Cheatham III, T. E.; Darden, T. A., Duke, R. E., Giese, T. J., Gohlke, H., Goetz, A. W., Homeyer, N. *Amber 2017*, University of California, San Francisco: San Francisco, 2017.

(8) Schrödinger. *Release 2017-4*: *Maestro*, Schrödinger, LLC: New York, NY, 2017.

(9) Marelius, J., Kolmodin, K., Feierberg, I., Aqvist, J. Q: a Molecular Dynamics Program for Free Energy Calculations and Empirical Valence Bond Simulations in Biomolecular Systems. *J. Mol. Graph. Model.* **1998**, *16*, 213-225.

(10) Bauer, P., Barrozo, A., Purg, M., Amrein, B. A., Esguerra, M., Wilson, P. B., Major, D. T., Aqvist, J., Kamerlin, S. C. L. Q6: A comprehensive toolkit for empirical valence bond and related free energy calculations. *Software X* **2018**, *7*, 388-395.
